# *Drosophila* Toxicogenomics: genetic variation and sexual dimorphism in susceptibility to 4-Methylimidazole

**DOI:** 10.1186/s40246-024-00689-3

**Published:** 2024-11-04

**Authors:** Katelynne M. Collins, Elisabeth Howansky, Sarah C. Macon-Foley, Maria E. Adonay, Vijay Shankar, Richard F. Lyman, Nestor Octavio Nazario-Yepiz, Jordyn K. Brooks, Rachel A. Lyman, Trudy F. C. Mackay, Robert R. H. Anholt

**Affiliations:** Center for Human Genetics, Department of Genetics and Biochemistry, Clemson University, Greenwood, SC 29646 USA

**Keywords:** Quantitative genetics, genome wide association analyses, Drosophila Genetic Reference Panel, sex-specific effects, sexually antagonistic effects, network associations with complex traits

## Abstract

**Background:**

4-methylimidazole is a ubiquitous and potentially carcinogenic environmental toxicant. Genetic factors that contribute to variation in susceptibility to its toxic effects are challenging to assess in human populations. We used the *Drosophila melanogaster* Genetic Reference Panel (DGRP), a living library of natural genetic variation, to identify genes with human orthologs associated with variation in susceptibility to 4-methylimidazole.

**Results:**

We screened 204 DGRP lines for survival following 24-hour exposure to 4-methylimidazole. We found extensive genetic variation for survival, with a broad sense heritability of 0.82; as well as genetic variation in sexual dimorphism, with a cross-sex genetic correlation of 0.59. Genome-wide association analyses identified a total of 241 candidate molecular polymorphisms in or near 273 unique genes associated with survival. These polymorphisms had either sex-specific or sex-antagonistic effects, and most had putative regulatory effects. We generated interaction networks using these candidate genes as inputs and computationally recruited genes with known physical or genetic interactions. The network genes were significantly over-represented for gene ontology terms involving all aspects of development (including nervous system development) and cellular and organismal functions as well as canonical signaling pathways, and most had human orthologs.

**Conclusions:**

The genetic basis of variation in sensitivity to acute exposure to 4-methylimidazole in Drosophila is attributable to variation in genes and networks of genes known for their effects on multiple developmental and cellular processes, including possible neurotoxicity. Given evolutionary conservation of the underlying genes and pathways, these insights may be applicable to humans.

**Supplementary Information:**

The online version contains supplementary material available at 10.1186/s40246-024-00689-3.

## Background

4-methylimidazole is a ubiquitous environmental toxicant used in the caramelization of food and beverages, including coffee and cola-type beverages, and can be formed through the Maillard reaction during cooking, roasting, and baking of food [1–3]. 4-methylimidazole is a possible carcinogen. Exposure to 4-methylimidazole has been reported to cause lung tumors in mice [4, 5], although subsequent analyses did not unequivocally support this conclusion [6]. Concentrations above 150 mg/mL affected viability of cultured rat bone marrow mesenchymal stem cells, but concentrations below those found in food and beverages (100 mg/mL) had no significant effect [7]. Feeding mice 4-methylimidazole resulted in a significant increase in chromosomal aberrations in bone marrow cells [8], and exposure to 4-methylimidazole in rats increased the incidence of mononuclear cell leukemia [4]. Genotoxic effects of 4-methylimidazole on human peripheral lymphocytes in cell culture have also been reported [9]. It is challenging to evaluate carcinogenic effects of 4-methylimidazole and variation in susceptibility to its potential carcinogenic or other toxic effects in human populations: dietary exposure is variable and cannot be accurately assessed, and exposure to multiple toxicants presents a further confounding factor. In addition to genetic heterogeneity and environmental factors unrelated to toxic exposure, the haplotype structure of the human genome [10] impedes precise mapping of variants and genes associated with toxicant-induced disease risk.

Comparative genomic studies using model organisms can provide insights into the genetic underpinnings that determine susceptibility to toxic exposure because fundamental biological pathways that respond to environmental toxic exposure are likely to be evolutionarily conserved across the phylogeny [11]. *Drosophila melanogaster* is an ideal model for genetic studies on toxic exposure [12]. Flies can be grown rapidly in large numbers, economically and without regulatory restrictions, in controlled genetic backgrounds with precise control of toxic exposure. We took advantage of extensive naturally occurring genetic variation in the inbred lines with complete annotated genome sequences of the *D. melanogaster* Genetic Reference Panel (DGRP) [13, 14]. Individuals within each DGRP line are nearly genetically identical, enabling repeated measurements on the same genotypes, thereby greatly increasing statistical power [13, 14]. In contrast to the structure of the human genome, linkage disequilibrium in Drosophila decays within a few hundred base pairs on average, which facilitates high-resolution mapping of polymorphic molecular variants associated with phenotypic variation [13, 14]. Furthermore, about 75% of human disease-associated genes have Drosophila orthologs, which enables translational inferences from the Drosophila model to human populations [15].

We show here that survival of DGRP lines following exposure to 4-methylimidazole is genetically variable and exhibits both sexual dimorphism and genetic variation in sexual dimorphism (sex-by-genotype interaction). We performed genome-wide association analyses and identified candidate genes and interaction networks with human orthologs that harbor molecular polymorphisms associated with variation in susceptibility to 4-methylimidazole.

## Methods

### Drosophila stocks and husbandry

Flies were maintained in 28.5 mm wide polypropylene vials (Genesee Scientific Cat. #32–120) on standard Bloomington cornmeal food (Bloomington Formulation, Nutri-Fly™, #66–112, Genesee Scientific) at 25 °C with a 12 h:12 h light and dark cycle. We have recently expanded the DGRP to 1,037 inbred, wild-derived, sequenced lines (DGRP3) from the same Raleigh, NC population from which the DGRP1 [13] and DGRP2 [14] lines were derived. All DGRP3 lines were derived by 20 generations of full-sib inbreeding of the progeny of wild-caught females. The extant 1,037 DGRP3 lines survived inbreeding and therefore some deleterious alleles present in the outbred population from which these lines were derived will have been lost. However, the DGRP3 still harbors extensive natural genetic diversity. The DGRP lines used in this study have 4,539,038 biallelic variants, of which 3,912,378 are single nucleotide polymorphisms (SNPs), 418,113 are deletions and 208,547 are insertions. The DGRP3 lines used in this study are listed in Additional File 1.

### 24-hour toxicity assay

4-methylimidazole (C₄H₆N₂) was purchased from Thermo Scientific Chemicals, Inc. A preliminary range-finding assessment was performed on Canton-S B (CSB) flies and validated with 14 maximally diverse DGRP2 lines using a modified version of a previously established protocol [16] to determine the concentration of 4-methylimidazole that resulted in 50% survival and maximal variation between DGRP lines after 24 h of exposure for females and males. After establishing optimal discriminating concentrations, we assayed 204 DGRP3 lines for susceptibility to 4-methylimidazole.

Two weeks before exposure, controlled adult density vials were set up using five female and five male flies for each DGRP3 line. Flies were transferred to a new vial 48 h later to encourage egg-laying and generate a larger population of progeny for the experiment. After another 48 h, the vials were cleared of adult flies. After seven days, fly progeny from each DGRP line were anesthetized using CO_2_ and sorted into groups of 15–20 same-sex flies. At three to five days post-eclosion and after a minimum recovery period of three hours from CO_2_ anesthesia, flies were food-deprived for 20 h in vials containing three pieces of 20 mm Whatman #1 filter paper (Cytiva Cat. #1001-020), wetted with 0.35 mL of distilled water. Subsequently, the flies were transferred into vials containing three pieces of 20 mm Whatman #1 filter paper and 0.35 mL of exposure solution consisting of 4-methylimidazole, yeast, sucrose, and distilled water, with four biological replicates of 15–20 individuals per sex. After 24 h of exposure, survival of each replicate was documented and averaged across all replicates. The assays of survival of DGRP lines and replicates were strictly randomized. The standard errors for all survival graphs were calculated as $\:\sqrt{p(1-p)/N}$, where $\:p$ is the proportion surviving pooled across all replicates and $\:N$ is the total number of individuals assessed in each line.

### Statistical analysis of sensitivity to 4-methylimidazole

The survival data were analyzed using the “PROC MIXED” command (Type III) in SAS™ Studio v3.8 software [17] according to the ANOVA model $\:Y=\mu\:+L+S+L\times\:S+\epsilon\:$, where $\:Y$ is the survival proportion, $\:\mu\:$ is the population mean, $\:L$ is the random effect of DGRP line, $\:S$ is the fixed effect of sex, $\:L\times\:S$ is the random effect of the Line by Sex interaction, and $\:\epsilon\:$ is the residual error. Reduced models of the form $\:Y=\mu\:+L+\epsilon\:$ were also performed for each sex. Note that we did not include a ‘no treatment control’, because 24-hour survival of young flies under these assay conditions is nearly 100%. The covariance parameter estimates derived from the SAS mixed model output for the full and reduced models were used to calculate the broad-sense heritability ($\:{H}^{2}$) and cross-sex genetic correlation ($\:{r}_{GS}$), respectively. We estimated broad sense heritability as $\:{H}^{2}=({\sigma\:}_{L}^{2}+{\sigma\:}_{L*S}^{2})/({\sigma\:}_{L}^{2}+{\sigma\:}_{L\times\:S}^{2}+{\sigma\:}_{\epsilon\:}^{2})$, where $\:{\sigma\:}^{2}$ denotes a variance component and the subscripts indicate the random effect terms. Cross-sex genetic correlations were calculated as $\:{r}_{GS}={\sigma\:}_{L}^{2}/\left({\sigma\:}_{LF}{\sigma\:}_{LM}\right)$, where $\:{\sigma\:}_{L}^{2}$ is the variance among lines from the full model, $\:{\sigma\:}_{LF}$ is the square root of the female among line variance, and $\:{\sigma\:}_{LM}$ is the square root of the male among line variance from the female and male reduced models, respectively [18].

### DNA sequencing

DNA was extracted from whole flies using the Zymo Research Quick-DNA Tissue/Insect Miniprep Kit. DNA was quantified using the Invitrogen Qubit 4 Fluorometer and Thermo Scientific NanoDrop™ 8000 Spectrophotometer. DNA libraries were prepared using a modified NEBNext ^®^ Ultra DNA Library Prep protocol. Libraries were quantified using the Invitrogen Qubit 4 Fluorometer and TapeStation 4150 System. All samples were sequenced to ~ 40X coverage using Illumina NovaSeq 6000 S1 Reagent Kits v1.5 and 2 × 150 chemistry (300 cycles).

### Variant calling

Raw sequencing data were filtered to remove short reads, low quality reads and bases, and trimmed to remove adapter sequences using the fastp pipeline [19]. High-quality sequence reads were aligned to the *D. melanogaster* reference genome (NCBI, release 6.13) with Burrows-Wheeler Aligner (BWA) v0.7.17 [20]. The alignments were sorted and indexed using Samtools (v1.10) [21] and locally realigned and marked for PCR duplicates with Genome Analysis Toolkit (GATK) v4.1 and Picard tools v2.21.7 before recalibrating base qualities with GATK [22]. Variant calling for each individual line was performed using the GATK haplotypecaller with the GVCF flag to generate genomic variant call format (VCF) files. Joint calling of individual genomic VCF files was performed using the GATK joint variant calling workflow with default parameters to generate a combined VCF file. The Joint Genotyping for Inbred Lines (JGIL) workflow [23] was used to account for expected homozygosity following 20 generations of full-sib inbreeding. The resulting variants were separated into single and multiple nucleotide and insertion/deletion molecular polymorphisms, then filtered to remove low-quality variants using standard filtering criteria recommended by the Broad Institute [24]. The VCF file containing high-quality variants was filtered to keep biallelic sites and to remove monomorphic and segregating sites. The filtered, combined VCF file was then converted into PLINK v1.9 format for genome-wide association testing [25].

### Covariate identification

Alignment files were used to identify the presence of the endosymbiont, *Wolbachia pipientis*. Reads that did not align to the *D. melanogaster* reference genome were aligned to two canonical *Wolbachia*-specific genes (*Wolbachia* Surface Protein and *w*Au ankyrin domain protein) using bbmap aligner v38.73 [26]. Successful alignment of more than 7,000 reads was considered as the criterium for positive infection state. The threshold was determined by constructing a frequency histogram for aligned reads across the two indicator genes.

Large inversions were identified by using processed alignment files as input for MANTA v1.6.0 [27]. Break end coordinates were converted to inversions using the *convertInversion.py* python script within the MANTA workflow. Individual batches of structural variant VCF files were split into single-sample VCF files and then merged using SURVIVOR (v1.0.7) [28] with a 1 kb margin for breakpoints and single aligner criteria. Only inversions with minor allele frequencies greater than 0.05 were used as covariates for association testing.

### Association testing

We performed genome-wide association (GWA) analyses using the pooled mean survival proportion of each line across all replicates. We performed separate GWA analyses for females, males, the average of females and males and the difference between the sexes. Preprocessing for association testing included filtering PLINK files and covariate files (*Wolbachia* infection status and common inversions) for lines included in the trait measurement. PLINK files were also filtered for sites based on a genotype missing rate of 0.1 and a minor allele frequency ≥ 0.015. Inclusion of covariates into the linear mixed effects modeling (LMM) for association testing was determined by fitting a generalized linear model for each covariate against the phenotype. Only covariates with *P*-values less than 0.1 were included in the LMM for the GWA analysis. The Genomic Relationship Matrix (GRM), calculated using GEMMA v0.98.5 [29], was used as a covariate to account for cryptic relatedness. The GWA analyses were performed using GEMMA, and statistical significance was calculated using the Wald test. The *β* coefficients from the Wald test are estimates of additive effects, calculated as half the difference between means of individuals with the major and minor allele genotypes [18]. Quantile-quantile (Q-Q) plots and Manhattan plots were generated in R using *qqman* library [30]. Polymorphisms with *P*-values ≤ 10^− 5^ were considered suggestive of association and used for variant annotation using ENSEMBL’s Variant Effect Predictor (VEP) v111 [31].

### Interaction network analysis

Genes associated with variants ± 5 kb of their transcription start and end sites were considered candidate genes associated with variation in survival following exposure to 4-methylimidazole. We mapped the candidate genes from the GWA analyses for females and the difference between the sexes onto a network of known genetic and physical associations (FlyBase, release FB2022_03) [32] using Cytoscape v3.9.0 [33]. Genes connected to at least two candidate genes were included in these networks. The MCODE algorithm v1.5.1 [34] was used to create subnetworks based on the topology of densely connected regions. Orthology was evaluated using the Drosophila RNAi Screening Center Integrative Ortholog Prediction Tool (DIOPT) v9.0 [35, 36]. If there was evidence that multiple human genes may be an ortholog of a gene from the inferred network, only the human gene with the highest DIOPT score was considered.

To test the statistical significance of the observed architecture for the interaction networks, the degree of connectivity of each interaction network was calculated and compared with a null distribution via Monte Carlo permutation tests ($\:P={n}_{X}/N$), where $\:P$ is the $\:P$-value of the significance, $\:{n}_{X}$ is the number of permutations where the expected is greater than the observed connectivity, and $\:N$ is the number of all permutations [37]. The null distribution was generated by randomly selecting numbers of genes equal to those found in the observed network and calculating the degree of connectivity. Kernel density estimation (KDE) was used to visualize the gaussian structure of the null distribution. We used 10,000 permutations to generate the null distribution for each network. The degree of connectivity for the observed and the null distributions were generated using the *igraph* library v1.4.2 in R.

### Gene set enrichment

We performed enrichment analyses for the Biological Processes, Cellular Component, and Molecular Function Gene Ontology sets as well as the FlyBase phenotype for classical alleles gene set. This was done using a Fisher’s Exact Test with a Benjamini-Hochberg false discovery rate (BH-FDR) correction (FDR < 0.05) in PANTHER [38].

## Results

### Variation in susceptibility to 4-methylimidazole in the DGRP

**Fig. 1:**
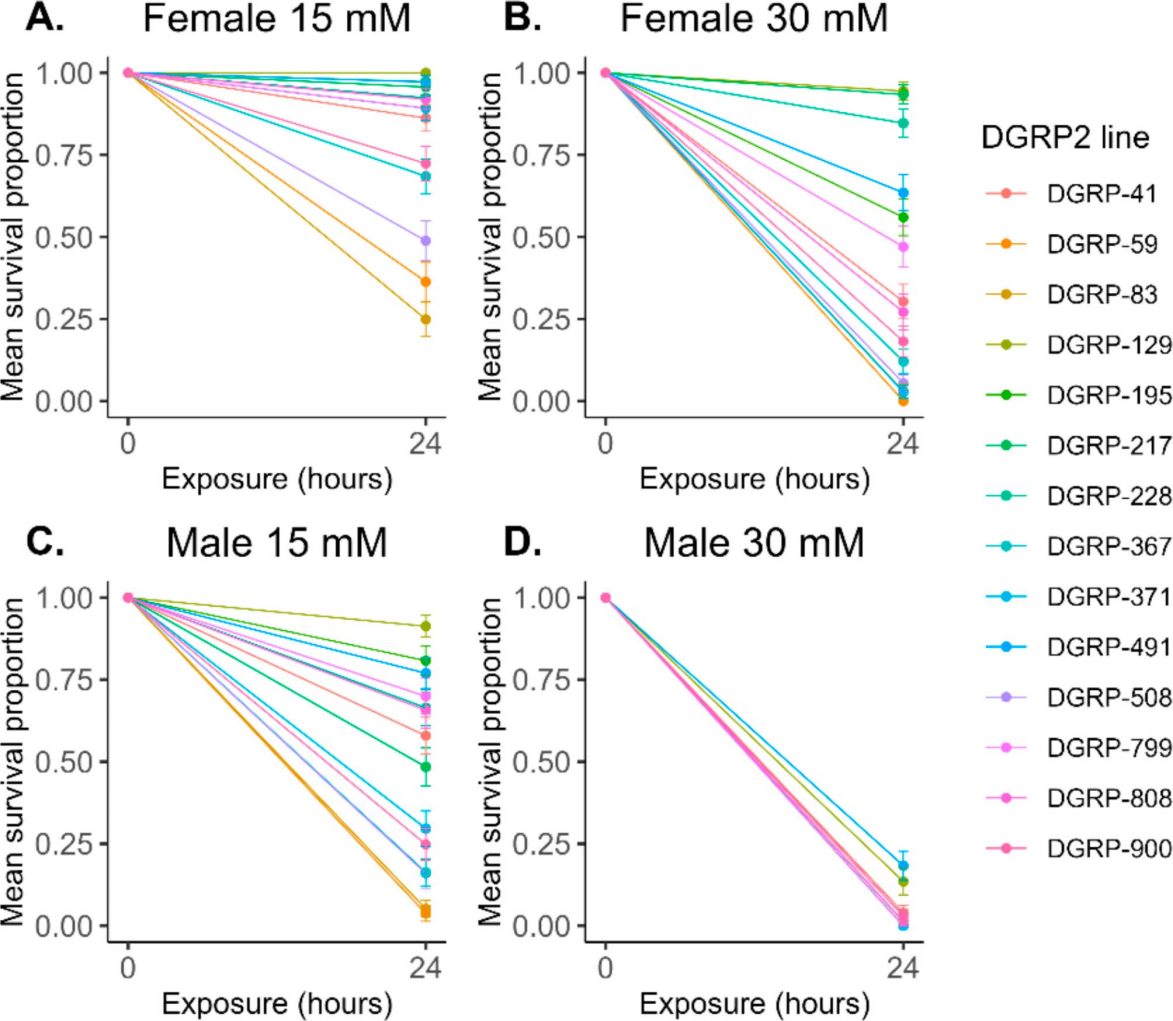
Optimally discriminating concentrations of 4-methylimidazole in the DGRP2. The proportion of flies surviving following 24-hour exposure to 15 mM and 30 mM 4-methylimidazole was quantified for 14 maximally diverse DGRP2 lines. Exposure to 30 mM for 24 h showed the greatest variation in female survival proportion (**A**, **B**) while exposure to 15 mM for 24 h showed the greatest variation for male survival proportion (**C**, **D**). Error bars represent standard errors of the mean

To determine a maximally discriminating concentration for survival of DGRP flies, we first measured the dose-response relationship for 4-methylimidazole-induced mortality in the standard laboratory CSB strain, separately for males and females. The LD50 concentrations were 15 mM for males and 30 mM for females, respectively, indicating sexual dimorphism in sensitivity to 4-methylimidazole exposure (Figure S1, Additional File 2 A, 2B). To assess whether the same concentrations would be optimally discriminating for an extensive toxicity screen of DGRP3 lines, we next measured 24-hour survival of 14 maximally divergent DGRP2 lines at 15 mM and 30 mM 4-methylimidazole for both sexes. We confirmed that 15 mM and 30 mM were indeed optimal concentrations for males and females, respectively, to reveal genetic variation for toxicant susceptibility across DGRP2 lines (Fig. 1, Additional File 3 A-C).

**Fig. 2:**
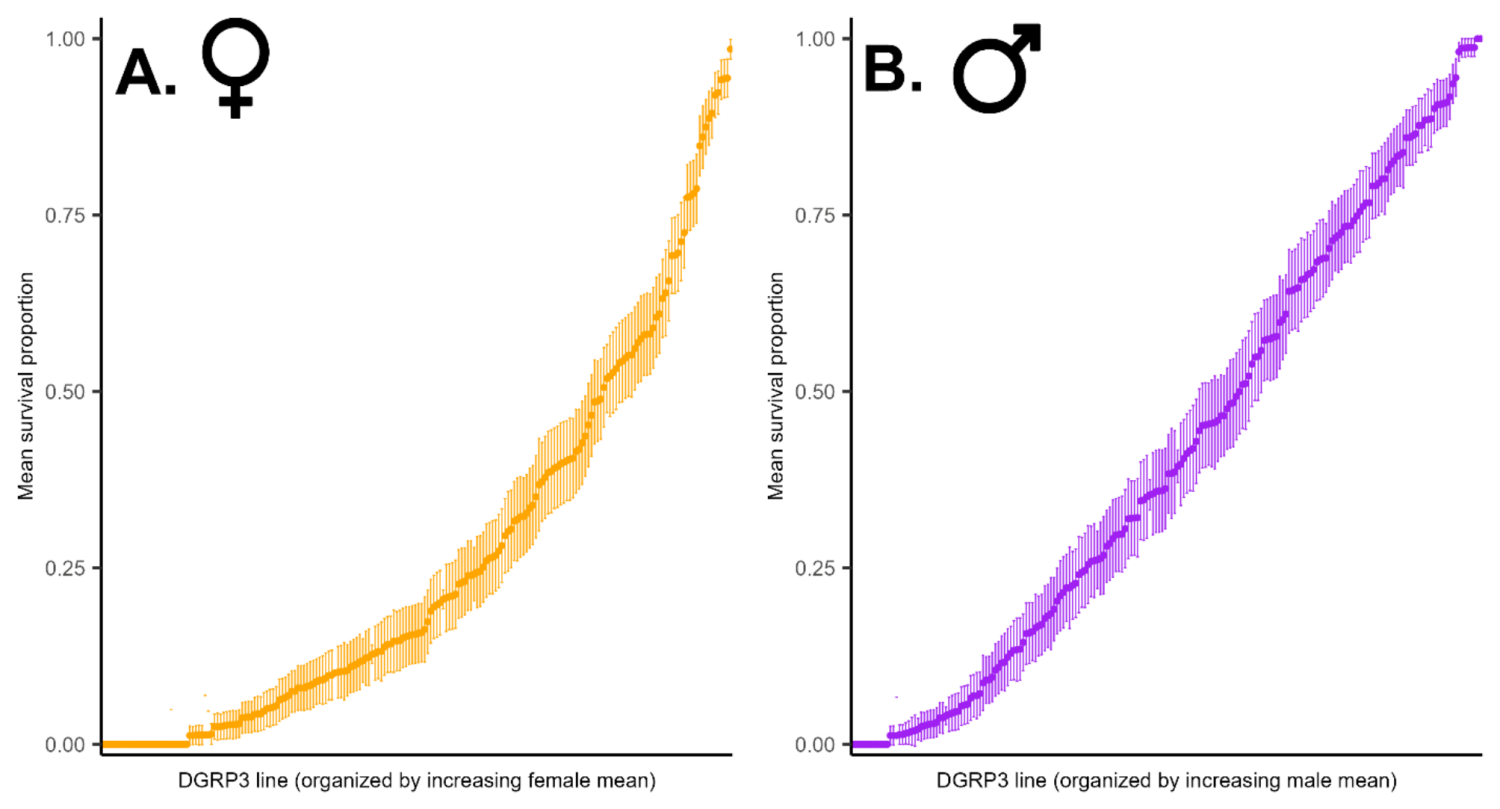
Variation in survival of females (**A**) and males (**B**) of 204 DGRP3 lines upon 24-hour exposure to 30 mM and 15 mM 4-methylimidazole, respectively. Datapoints are means of up to 80 individuals and error bars represent standard errors of the mean

Based on these data, we proceeded to screen 204 DGRP3 lines for 24-hour survival following exposure to 15 mM (males) and 30 mM (females) 4-methylimidazole, with up to 80 individuals per line per sex. We found extensive genetic variation in survival in both females and males, ranging from 0 to 100% in each sex (Fig. 2, Additional File 4 A-C). However, the rank order of DGRP3 lines for mean survival proportion was different between the sexes, indicating variation in sexual dimorphism for survival (Figure S2; Additional File 4).

**Fig. 3:**
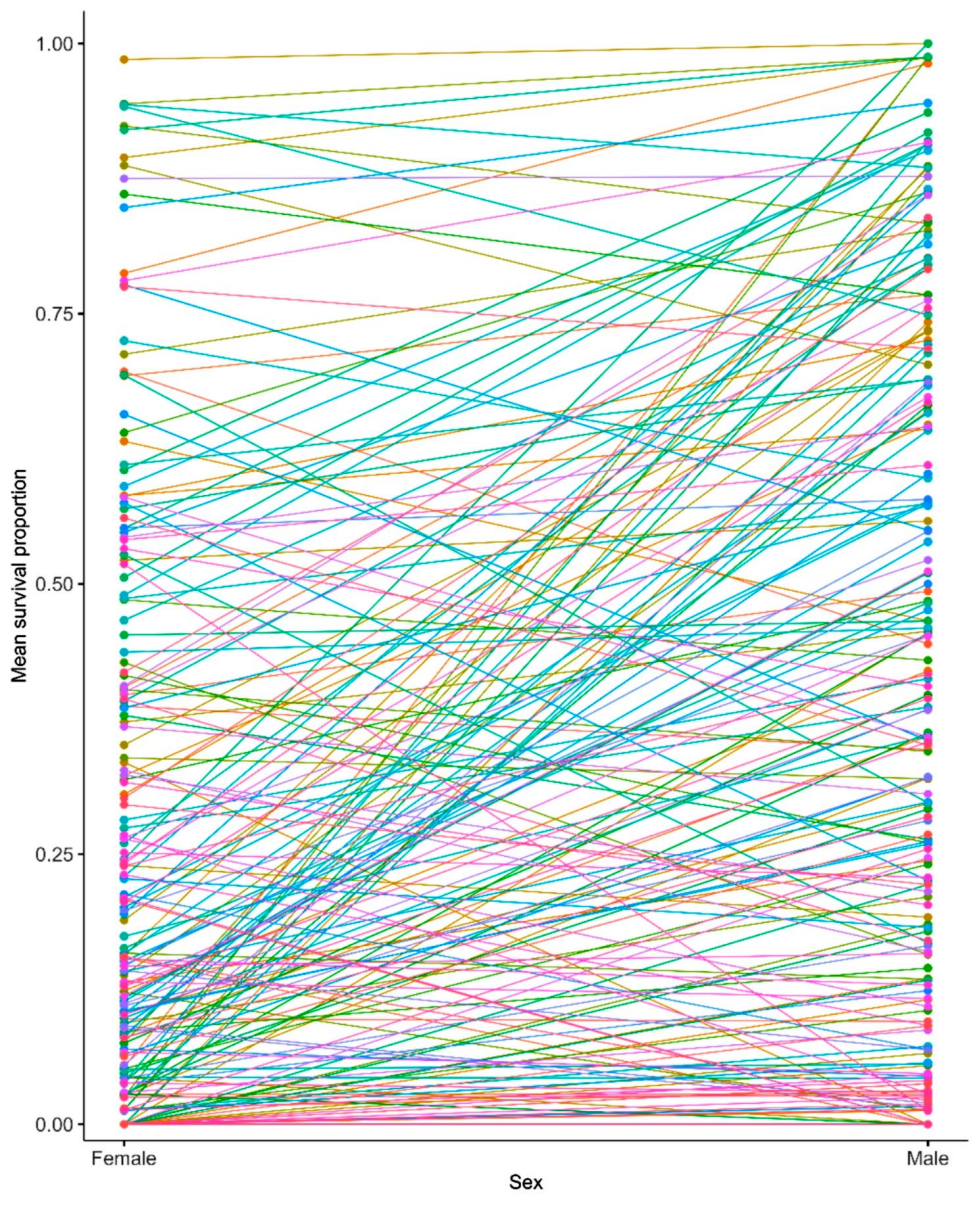
Reaction norms of female and male survival proportions for each DGRP3 line. The *x*-axis indicates females and males, and the *y*-axis indicates survival proportion

We partitioned variation in survival following 24-hour exposure to 4-methylimidazole into the contribution of sex, DGRP3 line and the sex by DGRP3 line interaction, and DGRP3 line separately for males and females (Additional file 4 C). Broad sense heritabilities ($\:{H}^{2}$) were $\:{H}^{2}$ = 0.80 for females and $\:{H}^{2}$ = 0.83 for males. The sex by line interaction variance component was significant and ~ 70% that of the among line variance component from the pooled analysis across

sexes (Additional File 4 C), indicating substantial genotype by sex interaction. The cross-sex genetic correlation ($\:{r}_{GS}$) was $\:{r}_{GS}$ = 0.588 ± 0.057 (SE). Since the cross-sex genetic correlation is significantly different from both 0 and 1, the genetic basis of variation in sensitivity to 4-methylimidazole is partially overlapping and partially distinct between males and females. The reaction norm plot of survival proportions of males and females of the same DGRP3 lines (Fig. 3) shows that for many lines, the responses to treatment with 4-methylimidazole are opposite in females and males.

### Genome-wide association analyses for variation in susceptibility to 4-methylimidazole

Given the significant genetic variation in susceptibility to 4-methylimidazole, we assessed associations of *Wolbachia pipientis* infection status, the karyotype of 31 polymorphic inversions and 2,514,186 segregating variants with allele frequencies greater than 0.015 with mean 24-hour survival following exposure to 4-methylimidazole in the 204 DGRP3 lines. We performed these analyses separately for females and males, for the average of the two sexes and for the difference (female – male) between the sexes.

Inversion 3L_P3173045_L13135796 *(In(3 L)P*) was significantly associated with 24-hour survival in females (*P* = 2.68 × 10^− 7^), males (*P* = 2.53 × 10^− 3^) and the average of females and males (*P* = 6.02 × 10^− 6^); and inversion 2R_P15391223_L4885174 (*In(2R)NS*) was associated with the difference between males and females in 24-hour survival (*p* = 7.45 × 10^− 3^) (Additional File 5). All covariates with *P*-values < 0.1 for association in each analysis were included in the GWA study for that respective analysis (Additional File 5).

**Fig. 4:**
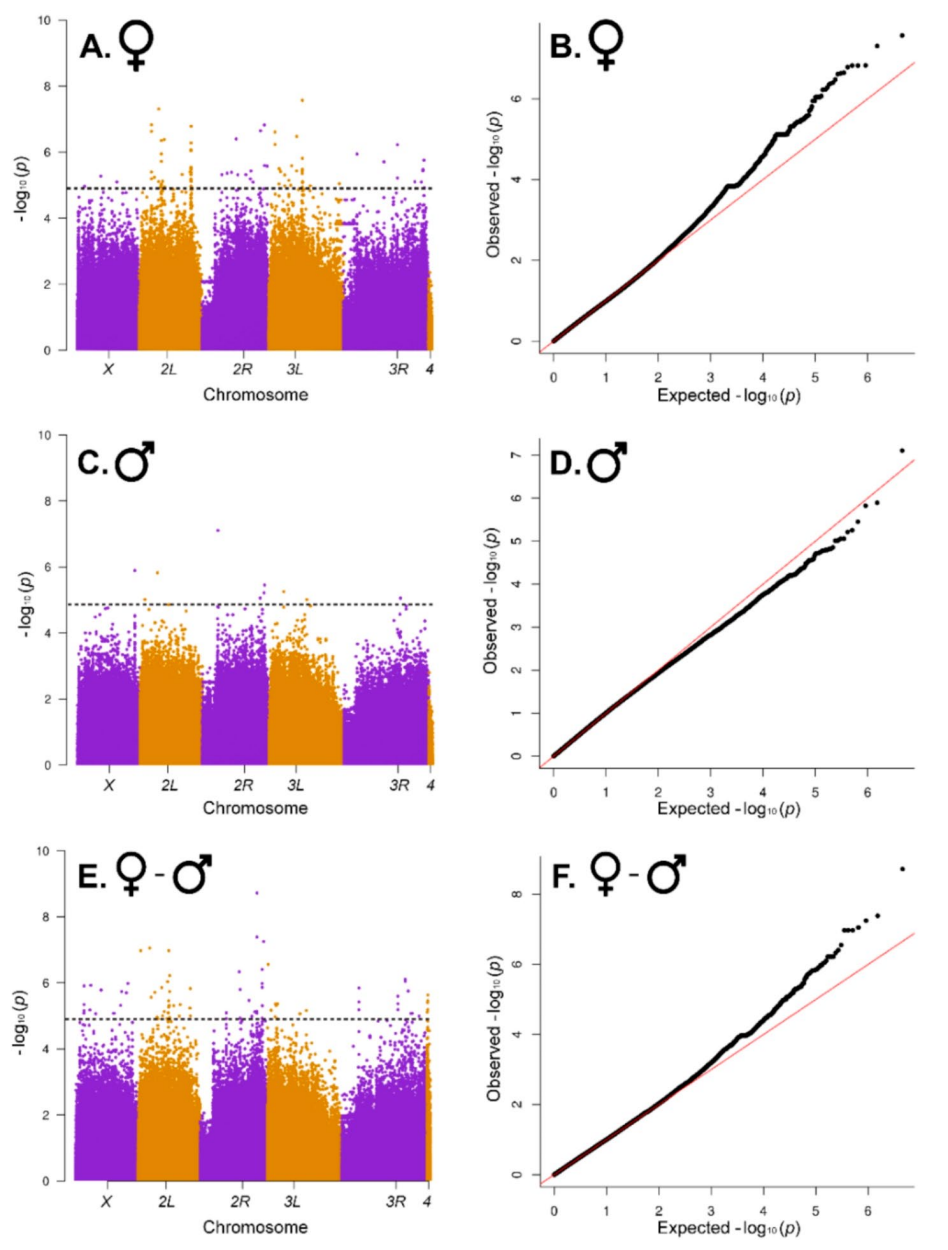
Manhattan and QQ plots for genome-wide associations of molecular polymorphisms with variation in sensitivity to 4-methylimidazole for females (**A**, **B**), males (**C**, **D**), and the difference in the survival proportion of females and males (**E**, **F**). On the Manhattan plots (**A**, **C**, **E**), the *x*-axes show the Drosophila chromosome arms, and the horizontal dotted line designates the statistical threshold of *P* < (‒log_10_(*P*) = 5). The red diagonal line in the QQ plots (**B**, **D**, **F**) is the expected *P*-value under the null hypothesis of no significant associations; the black points are the observed *P*-values

We used a nominal *P*-value of *P* < 10^− 5^ to identify candidate variants and genes [13, 14] in the four GWA analyses. The female dataset had many observed *P*-values below this threshold (Fig. 4A and B), but the male dataset did not (Fig. 4C and D). The GWA analysis for the average of males and females also showed few observed *P*-values ≤ 10^− 5^ (Supplementary Figure S3); in contrast, the GWA analysis for the difference between males and females had substantially more associations at *P*-values ≤ 10^− 5^ (Fig. 4E and F). We identified 134 candidate molecular polymorphisms in or near (within 5 kb of the gene body) 89 unique genes for females, 10 molecular polymorphisms in or near 15 genes for males, 31 molecular polymorphisms in or near 32 genes for the average of females and males, and 85 molecular polymorphisms in or near 157 genes for the difference of females and males (Additional File 6 A). In total, we identified 241 molecular polymorphisms (211 single nucleotide polymorphisms (SNPs), and 30 insertion/deletion polymorphisms) in or near 273 genes (Additional File 6 A). Variants with lower minor allele frequencies tended to have larger effects than those with intermediate minor allele frequencies, as has been observed previously [13, 14].

One SNP (*2R*_21319198_T_C) was significant following a Bonferroni correction for multiple tests (*P* < 1.99 × 10^− 8^). This SNP could potentially affect three genes: it is located 842 bp downstream of *CG30394*; in the 3’ UTR of *CG33655*; and is a synonymous polymorphism in *CG9485*. Four other SNPs had relatively low *P*-values, although they did not reach Bonferroni level significance. *3L*_12593961_A_G (*P* = 2.71 × 10^− 8^) is in an intron of *ara*; *2L*_7230508_G_T (*P* = 4.92 × 10^− 8^) is a synonymous variant in *Ndae*; and *2L*_4374432_G_A (*P* = 8.87 × 10^− 8^) is in an intron of *Traf4*. *2R*_21329761_A_C (*P* = 4.10 × 10^− 8^) is localized to an intron of *dom* and an exon of *snoRNA: Psi28S-3378*, and is within 1 kb upstream of five other snoRNAs located in the same *dom* intron (Additional File 6). *CG30394* is involved in transmembrane transport of amino acids; *CG9485* is involved in the biosynthesis and catabolism of glycogen, and the function of *CG33655* is not known. *ara* (*araucan*) is a transcriptional regulator involved in the morphogenesis, development and differentiation of neurons, the compound eye, muscles and wings. *dom* (*domino*) is also a transcriptional regulator that affects chromatin remodeling and alternative mRNA splicing as well as developmental processes. *Ndae* (*Na*^*+*^*-driven anion exchanger 1*) regulates cellular pH and the transmembrane transport of chloride, protons and sodium ions. *Traf4* (*TNF-receptor-associated factor 4*) is a pleiotropic gene involved in multiple signaling pathways and developmental processes (Additional File 6B).

Most of the remaining candidate polymorphisms were also intronic, located up- or down-stream of candidate genes, or greater than 5 kb from the nearest gene (intergenic), and hence play putative regulatory roles associated with variation in viability following 24-hour exposure to 4-methylimidazole. These polymorphisms potentially affect multiple genes, highlighting the difficulty of determining causality in the densely packed Drosophila genome. The candidate genes included nine antisense RNAs, 27 long non-coding RNAs and 15 small nucleolar RNAs, which also regulate gene expression (Additional File 6 A). Indeed, 14 of the remaining 222 genes in or near significant molecular polymorphisms were annotated to affect transcriptional regulation [38] and 11 affect post-transcriptional processes (Additional File 6B). A total of 45 candidate genes affect nervous system development, morphogenesis and function, implicating natural genetic variation in susceptibility to neurotoxicity as a possible contributor to the variation in viability following exposure to 4-methylimidazole. Among these genes is *bruchpilot* (*brp*), which encodes a gene product that contributes to calcium channel clustering at presynaptic active zones [39, 40]. Perhaps surprisingly, 47 candidate genes have pleiotropic effects on development of many tissues and organ systems, implicating these genes in adult functions. In addition, two candidate genes affect DNA repair, consistent with 4-methylimidazole-induced chromosomal aberrations found in mice [8]; 14 affect reproduction; six affect immune/defense responses; 17 are involved in canonical signaling pathways; 13 affect metabolism and four are involved in detoxification (Additional File 6B). A total of 106 of the candidate genes had no previous Biological Process annotation. Of the 273 candidate genes, 129 had at least one human ortholog with a DIOPT [35, 36] score ≥ 3 (Additional File 6 C).

There was no overlap between candidate molecular polymorphisms detected in the female, male and sex difference GWA analyses (Additional File 6D). This is consistent with the ANOVA results that show a significant sex by line variance component and hence genotype by sex interaction for survival following exposure to 4-methylimidazole (Additional File 6D). The different significant molecular polymorphisms detected in separate GWA analyses of females and males have sex-specific effects. The significant molecular polymorphisms detected in the GWA analysis of the difference between females and males have sex-antagonistic effects. Although not significant in either the female or male GWA analyses, these molecular polymorphisms have opposite effects in the two sexes (Additional File 6D). There is some overlap between molecular polymorphisms detected in the single sex analyses and the average of the two sexes (Additional File 6D). These are technically sex-specific effects, since they are only significant in females or males, but the effect in the other sex was in the same direction as the significant sex, such that the average crossed the significance threshold (Additional File 6D).

### Interaction networks associated with variation in susceptibility to 4-methylimidazole

**Fig. 5:**
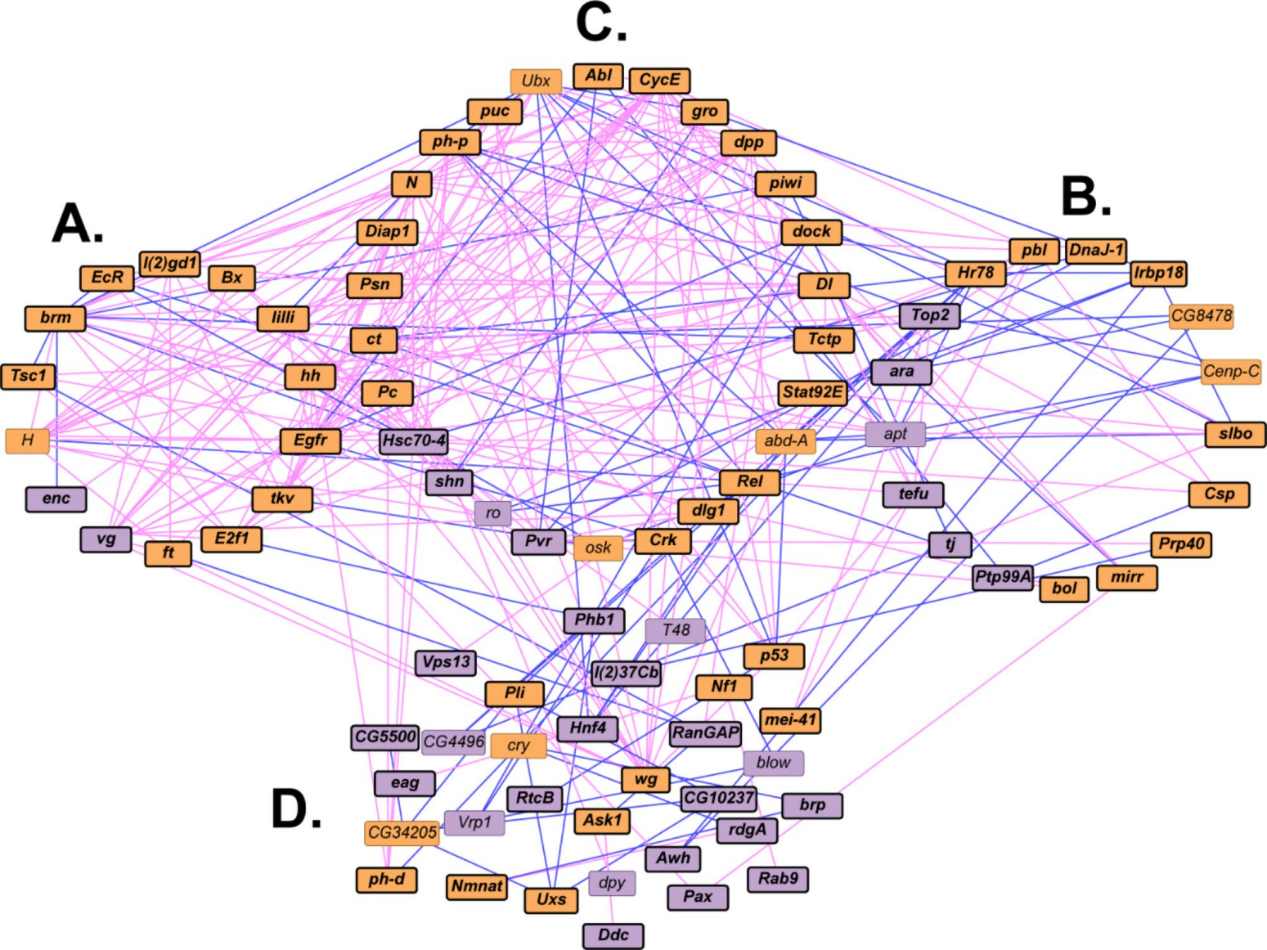
An interaction network based on candidate genes from the female GWA analysis consisting of two modules (**A**, **B**), the overlapping genes between them (**C**), and unclustered genes (**D**). Candidate genes identified in the GWA analysis are indicated as purple boxes and computationally recruited genes are indicated as orange boxes. Bold font and black borders show genes with strong human orthologs (DIOPT score ≥ 7). Pink edges designate genetic interactions and blue edges indicate physical interactions

**Fig. 6:**
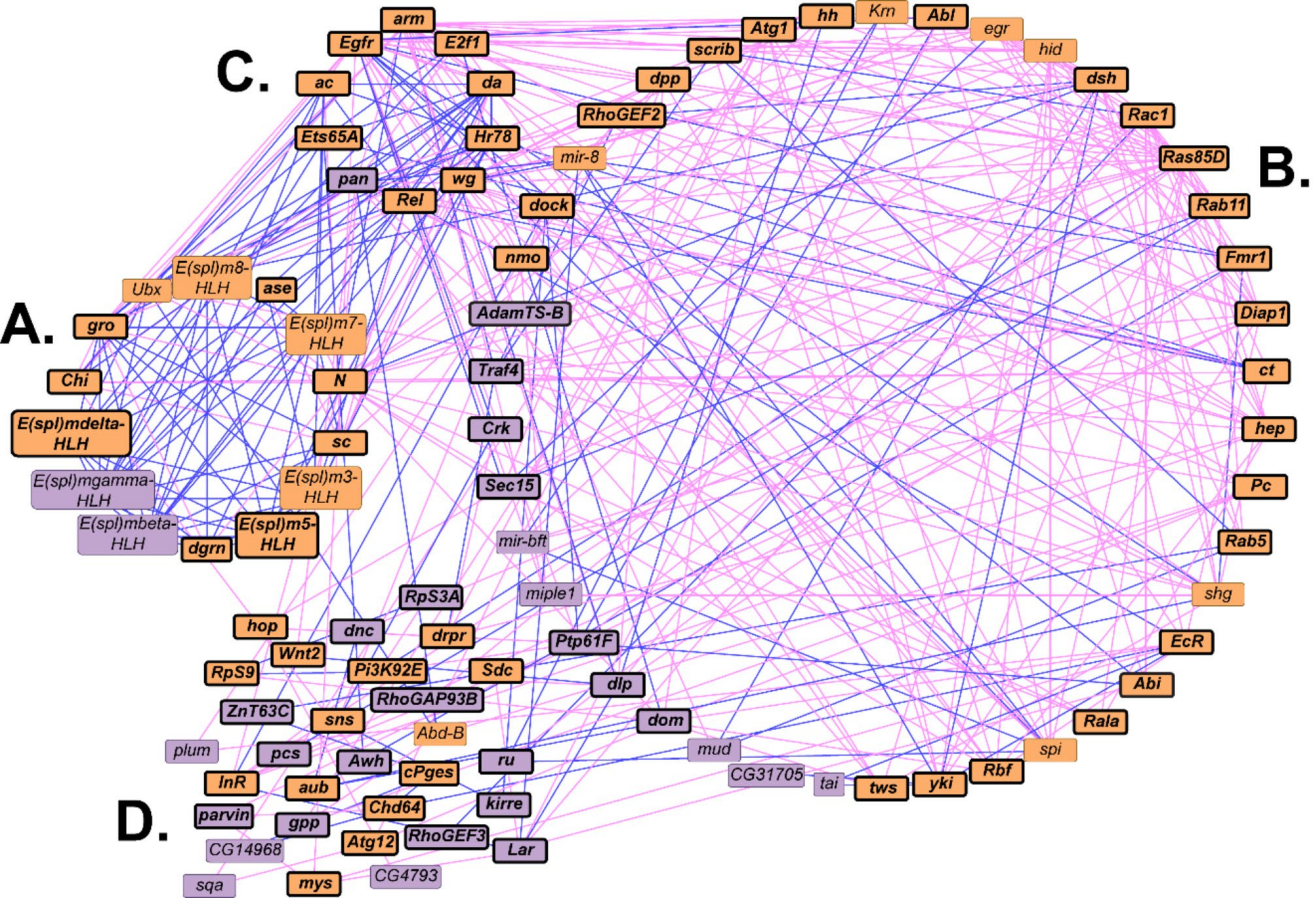
An interaction network based on candidate genes from the difference GWA analysis consisting of two modules (**A**, **B**), the overlapping genes between them (**C**), and several unclustered genes (**D**). Candidate genes identified through genome-wide association analysis are indicated as purple boxes; computationally recruited genes are indicated as orange boxes. Bold font and black borders show genes with human orthologs (DIOPT score ≥ 7). Pink edges designate genetic interactions; blue edges indicate physical interactions

To explore functional contexts of the candidate genes from the GWA analyses, we constructed networks in which we recruited genes with known physical or genetic interactions for females (Fig. 5) and for the difference between females and males (Fig. 6). There were not sufficient candidate genes from the male and sex average GWA analyses to perform these analyses. The female network contains 88 genes, of which 32 are candidate genes from the GWA analysis (Fig. 5. Additional File 7 A). The network consists of two modules comprised of genes that distinguish the modules (Fig. 5A and B) and that overlap between them (Fig. 5C), and a set of unclustered genes. The sex-difference network consists of 96 genes, 31 of which were candidate genes from the GWA analysis for variation in survival (Fig. 6, Additional File 7 A). This network has a similar architecture to that derived from the female GWA, with two overlapping modules consisting of genes that are unique to each module (Fig. 6A and B), genes that are in common between the two modules (Fig. 6C), and unclustered genes (Fig. 6D). Although there is no overlap of molecular polymorphisms in the female and sex-average GWA analyses, a total of 17 genes overlap between the computationally recruited networks (Additional File 7 A).

We performed gene set enrichment analyses for the Biological Process Gene Ontology (GO) and PANTHER pathway categories [38] separately for the networks derived from the female (Additional File 7B) and sex difference (Additional File 7 C) GWA analyses. Although only 17 genes overlapped between these analyses, many GO and pathway terms were significantly over-represented in both analyses ((FDR < 0.05; Additional File 7D). These terms include the development, morphogenesis and differentiation of antennae, appendages (halteres, legs, wings), the digestive system, eyes, gametes, gonads, growth in general, heart, imaginal discs, muscles, Malpighian tubules, the nervous system and the open tracheal system. Over-represented terms common to both networks impinge on multiple cellular and organismal functions: apoptosis, asymmetric cell division, behaviors, biosynthesis, cell cycle, cell division, cellular responses to stimuli and stresses, circadian rhythm, cognition, DNA damage response, gene expression, hemopoiesis, immunity, learning, lifespan, locomotion, metabolism, memory, reproduction and sex differentiation (Additional File 7D). The genes encoding cadherin, Epidermal Growth Factor, Notch, p53 and Wingless were also over-represented in both networks. The network genes had a combined total of 808 human orthologs (DIOPT score ≥ 3) and 323 strong human orthologs (DIOPT score ≥ 7) (Additional File 7E).

We used a Monte Carlo permutation procedure to determine the statistical significance of the observed connectivity in the interaction networks constructed from our candidate genes. We compared the observed degree of connectivity (female survival proportion = 6.43; differences in survival proportions = 8.21) to a null distribution generated by repeated random sampling of subnetworks (10,000 permutations) from the FlyBase database network of known genetic and physical associations, with the number of genes in the subnetworks constrained to the number of genes in observed interaction networks. This procedure was performed separately for each interaction network. The observed connectivity indices for the interaction networks constructed from the candidate genes associated with female survival proportions (Supplementary Fig. 4A) and differences in survival proportions (Supplementary Fig. 4B) were found to be strongly statistically significant at *P* < 0.0001. This indicates that both networks are densely interconnected and unlikely to be random.

### Comparison of protein-protein interactions between *Drosophila* and humans

To identify shared protein-protein interactions (PPI) between *Drosophila* and human data, we isolated the subnetwork of genes with strong human orthologs from *Drosophila* FlyBase interactions and used this gene set to filter the human PPI network from STRING database [41] using Cytoscape. To identify and organize the common edges between these two networks, we used the DyNet plugin [42] for Cytoscape. We identified 26 interactions shared with the human PPI network from STRING database, out of a total of 107 edges in the PPI FlyBase network constructed from the GWA analysis of differences in survival proportions (Supplementary Fig. 5). Similarly, we identified 9 interactions shared with the human PPI network, out of a total of 56 edges in the PPI FlyBase network constructed from the GWA analysis of female survival proportions (Supplementary Fig. 6). This results in an edge correctness (percentage of edges shared) of 24.3% and 16.1% for the two network comparisons.

## Discussion

We took advantage of natural variation in 204 inbred, wild-derived DGRP3 lines to identify molecular polymorphisms and their proximate candidate genes associated with variation in survival following 24-hour acute exposure to 4-methylimidazole. Our initial observations using the Canton-S B strain showed that sensitivity to 4-methylimidazole exposure is sexually dimorphic, with greater sensitivity in males than females. Therefore, we chose a lower concentration of 4-methylimidazole to assess variation in DGRP3 males (15 mM) than females (30 mM), since these concentrations maximized the variation in survival among 14 DGRP2 lines [13, 14]. We calculate that the filter paper on which the flies are exposed to the toxicant have been impregnated with about 430 ng of 4-methylimidazole for males and 860 ng for females. Given that male flies weigh on average 0.7 mg and females weigh on average 1 mg and given that only a fraction of the toxicant on the filter paper is consumed during the assay, we estimate that the exposure dose is well within the range of exposures used in previous studies on mice [4, 8] and several orders of magnitude lower than the concentrations administered to human peripheral lymphocytes in vitro [9].

The broad sense heritability of sensitivity to 4-methylimidazole exposure is high: $\:{H}^{2}$ = 0.82 averaged across females and males and similar for each sex separately. Therefore, the concentrations of 4-methylimidazole used in the larger screen indeed maximized variation in survival among genotypes within each sex, although the mean survival of males was greater than that of females. We also observed substantial genetic variation in sexual dimorphism, i.e., the difference in sensitivity between females and males was not constant across DGRP3 lines but varied in magnitude and even direction. Many DGRP3 genotypes showed lower average survival of males following exposure to 4-methylimidazole than females, despite the lower dose. The cross-sex genetic correlation was $\:{r}_{GS}$ = 0.588, suggesting a partially overlapping and partially distinct genetic basis of sensitivity to 4-methylimidazole between females and males. Thus, sensitivity to 4-methylimidazole at doses where variation among DGRP lines is maximized for each sex is a sex-specific trait, or survival at different concentrations may be different traits independent of sex. Variation in sexual dimorphism is observed for all quantitative traits assessed in the DGRP [43], including survival following exposure to the same concentrations of toxic heavy metals for each sex [44, 45], suggesting true sex-specificity of the genetic basis of sensitivity to 4-methylimidazole. Sex differences in sensitivity to environmental toxicants have also been documented extensively in human populations [46–48]. However, such studies do not address variation in sexual dimorphism, i.e., genetic background dependence in the magnitude/direction of sexual dimorphism.

We performed GWA analyses of survival following exposure to 4-methylimidazole for females, males, and the average and difference of the two sexes. We chose *P* < 10^− 5^ as our reporting significance threshold for association of molecular variants with survival based on quantile-quantile plots from the GWA analyses for females and the difference between females and males, which showed an excess of ‒log_10_*P*-values above 5. In contrast, there were few associations above this threshold in the male and sex-average GWA analyses. Consistent with our inferences from the quantitative genetic analyses of genetic variation in sensitivity to 4-methylimidazole, all molecular polymorphisms associated with variation in survival had sex-specific effects in females and males (i.e., were only significant in one sex). Molecular polymorphisms associated with variation in the difference between female and male survival all had opposite effects in females and males (i.e., were sex-antagonistic). The mechanisms that give rise to sex differences and variation in the magnitude/direction of difference between the sexes for complex traits are not understood. Such differences cannot be explained only due to size differences between Drosophila males and females but could conceivably reflect subtle differences in neural connectivity resulting from sex-biased gene expression during neural development. Sex-specific and -antagonistic effects are common for molecular polymorphisms associated with variation in *D. melanogaster* life history traits and may account for their persistence in natural populations [49]. Therefore, the genetic basis of naturally occurring variation in fitness is different in the two sexes. In addition, GWA analyses that pool across sexes or only assess a single sex can give misleading and/or incomplete results.

Several themes emerge from the GWA analyses of survival following exposure to 4-methylimidazole. First, sensitivity to this toxin is genetically complex, with 273 implicated candidate genes. While a few candidate genes (*Cyp9c1*, *GstE11*, *MtnC*, *ZnT63C*) have been associated with cellular detoxification of xenobiotics, as might be expected, the majority are associated with diverse biological processes. Chief among these are genes involved with the development, differentiation and function of the central and peripheral nervous systems, consistent with a neurotoxic effect of 4-methylimidazole. Candidate genes were also associated with DNA repair, reproduction, immune/defense responses, and metabolism. Many of the candidate genes were annotated based on their pleiotropic effects on the development, differentiation and morphogenesis of diverse tissues and organ systems, an observation made previously from GWA analyses of the effects of lead and cadmium on survival, development time and behavior [44, 45] and for Drosophila quantitative traits in general [43]. This suggests natural genetic variation in developmental processes that gives rise to variation in structure and function of relevant cell types, tissues and organs causes genetic variation in adult quantitative traits. Second, many candidate genes have not been previously associated with a biological process; this study suggests they are novel genes to functionally validate for their role in sensitivity to 4-methylimidazole and other toxins. Third, most of the candidate molecular polymorphisms and genes putatively exert their effects via a regulatory mechanism. Most molecular polymorphisms are up- or down-stream of candidate genes or intronic; and candidate genes include regulatory non-coding genes (antisense RNAs, long non-coding RNAs, small nucleolar RNAs); and genes that affect transcriptional and post-transcriptional regulation. Fourth, many candidate genes have human orthologs.

We further assessed the functional context of genes in the female and sex-difference GWA analyses by placing them into statistically significant genetic and physical interaction networks, while computationally recruiting additional genes in the network not implicated by the GWA analyses but which interacted with at least two GWA candidate genes. Although no genes were in common to these GWA analyses, 17 genes mapped to both networks (*Abl*, *Awh*, *Crk*, *ct*, *Diap1*, *dock*, *dpp*, *E2f1*, *EcR*, *Egfr*, *gro*, *hh*, *Hr78*, *N*, *Rel*, *Ubx*, *wg*). These genes are highly pleiotropic and play key roles in the development, differentiation and morphogenesis of the nervous system (*Abl*, *ct*, *N*), epithelium (*Abl*), wing (*Awh*, *ct*,, *E2f1*, *N*), abdomen (*Awh*), muscle (*ct*, *N*), oocyte (*ct*, *N*), actin cytoskeleton (*dock*), the open tracheal system (*Hr78*), the eye (*N*) and segmental appendages (*N*) [32]. Others play more general roles in growth regulation, patterning and cell fate (*dpp*, *E2f1*, *Egfr*, *hh*, *Ubx*,* wg*), apoptosis (*Diap1*), immunity (*Rel*) and many other biological processes [32]. The human ortholog of *Abl* tyrosine kinase (*ABL1*) is associated with chronic myeloid leukemia and the human ortholog of *Crk* (*CRK*) is a proto-oncogene [32], which could plausibly be relevant to potential carcinogenic effects of 4-methylimidazole [4, 8, 9]. The female and sex-difference networks were significantly over-represented for biological process gene ontology terms involving all aspects of development and cellular and organismal functions as well as canonical signaling pathways. Most genes in both networks have strong human orthologs (DIOPT scores ≥ 7).

Future studies could further corroborate our results through out-of-sample validation. The screen could be repeated by selecting DGRP3 lines from the 833 untested lines that are fixed for alternative alleles for each polymorphism of interest and testing the effect on susceptibility to 4-methylimidazole for these lines in which the focal alleles are homozygous in otherwise randomized genetic backgrounds [50, 51]. These studies are, however, outside the scope of the current study.

## Conclusions

The DGRP can be used to implement comparative genomics strategies to gain translational insights in the genetic underpinnings that determine variation in susceptibility to environmental toxicants, as shown in this study for 4-methylimidazole. Based on evolutionary conservation of fundamental biological processes and orthologies between Drosophila genes and their human counterparts, insights obtained from the Drosophila model can be used to infer responses to toxic exposure in human populations. Our data indicate that the genetic basis of variation in adult sensitivity to acute exposure to 4-methylimidazole is attributable to naturally occurring regulatory variation in genes and networks of genes known for their effects on multiple developmental and cellular processes, including possible neurotoxicity.

## Electronic Supplementary Material

Below is the link to the electronic supplementary material.

Supplementary Material 1

Supplementary Material 2

Supplementary Material 3

Supplementary Material 4

Supplementary Material 5

Supplementary Material 6

Supplementary Material 7

Supplementary Material 8

Supplementary Material 9

Supplementary Material 10

Supplementary Material 11

Supplementary Material 12

Supplementary Material 13

## Data Availability

All extant DGRP2 and DGRP3 lines are available from the Bloomington Drosophila Stock Center at the University of Indiana, Bloomington, IN. The aligned sequence data of the DGRP3 lines used in this study are available from the National Center for Biotechnology Information (NCBI) Short Read Archives (SRA) under BioProject PRJNA1139241. Input data, code for the Monte Carlo Permutation Procedure, and R visualization scripts are available on GitHub at https://github.com/amalgamaria/droso_toxicogenomics-genetic_variation_and_sexual_dimorphism_in_susceptibility_to_4_methylimidazole. Input data are also given in the Additional Files. The GWA analysis pipeline is available on GitHub at https://github.com/vshanka23/dgrp_gwas_final.
