# Supplementary figures and images for: Drosophila Toxicogenomics: genetic variation and sexual dimorphism in susceptibility to 4-Methylimidazole
Source: Hum Genomics. 2024 Nov 4;18:119. doi: 10.1186/s40246-024-00689-3 (PMC11533318; doi:10.1186/s40246-024-00689-3)

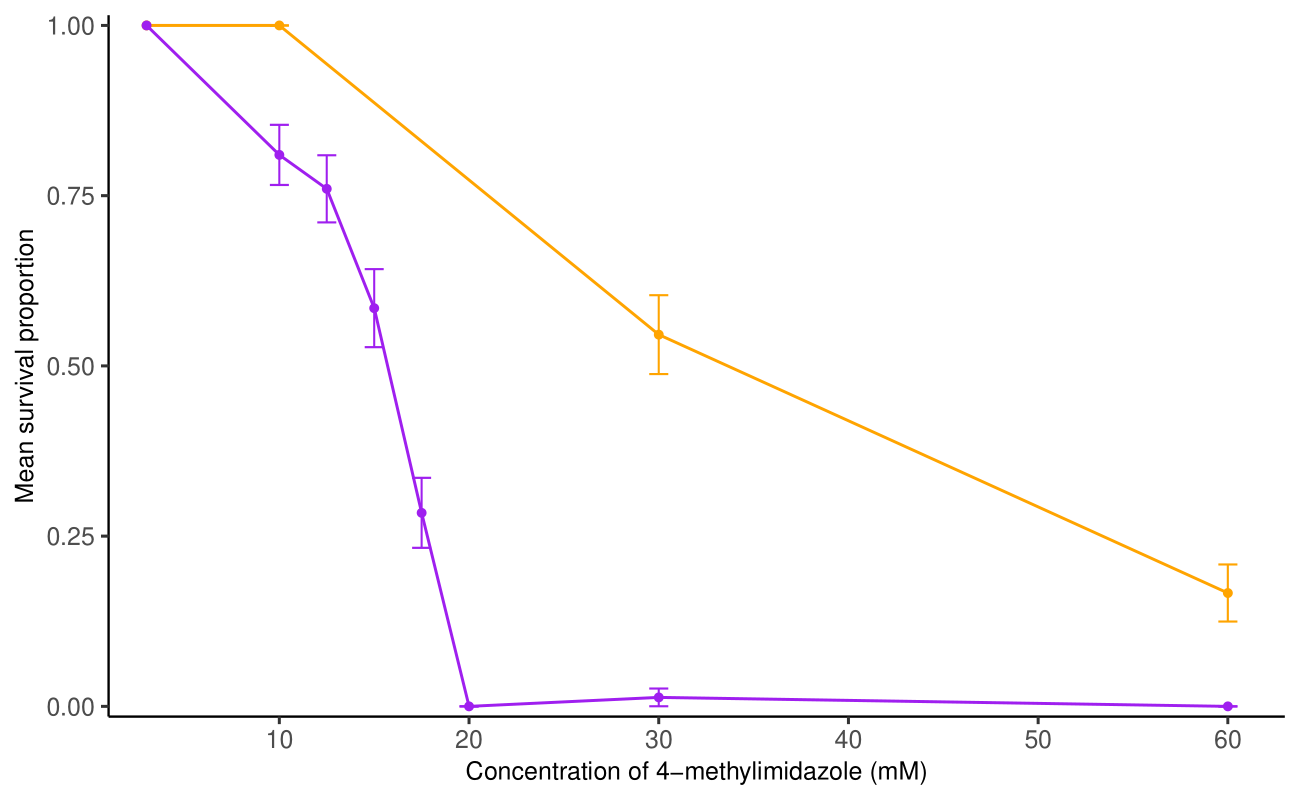

Supplement: Supplementary file 1 — Supplementary Material 1 [file 40246_2024_689_MOESM1_ESM.tif]

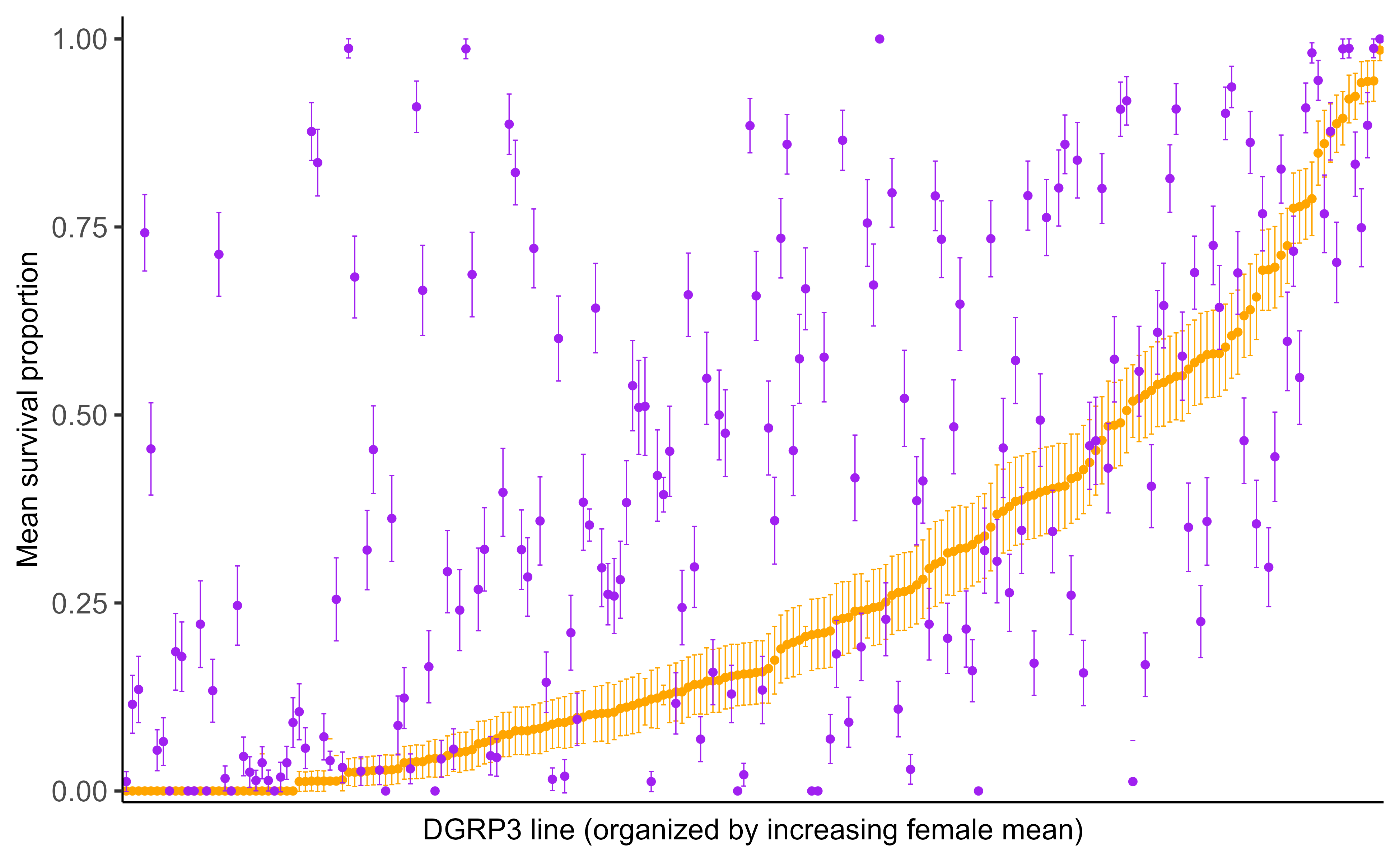

Supplement: Supplementary file 2 — Supplementary Material 2 [file 40246_2024_689_MOESM2_ESM.tif]

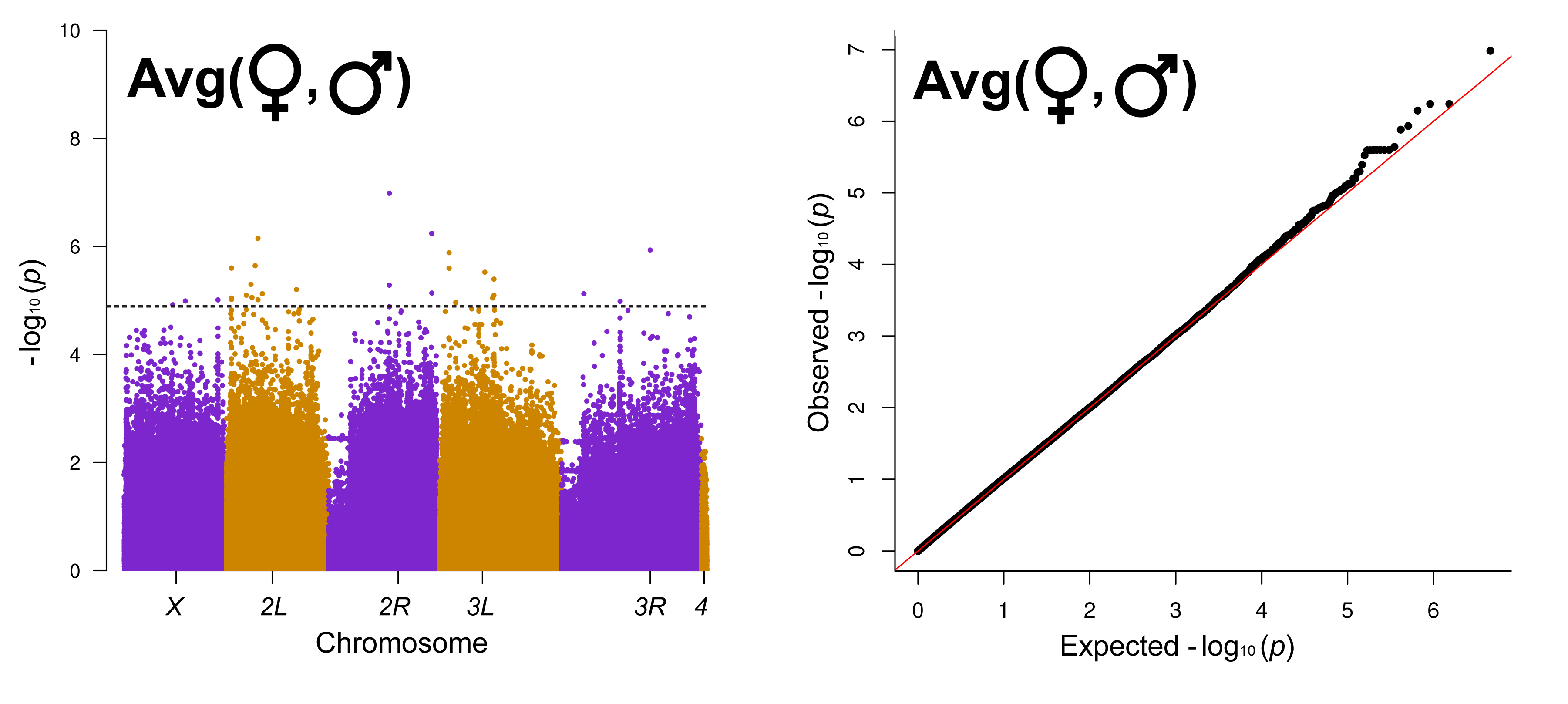

Supplement: Supplementary file 3 — Supplementary Material 3 [file 40246_2024_689_MOESM3_ESM.tif]

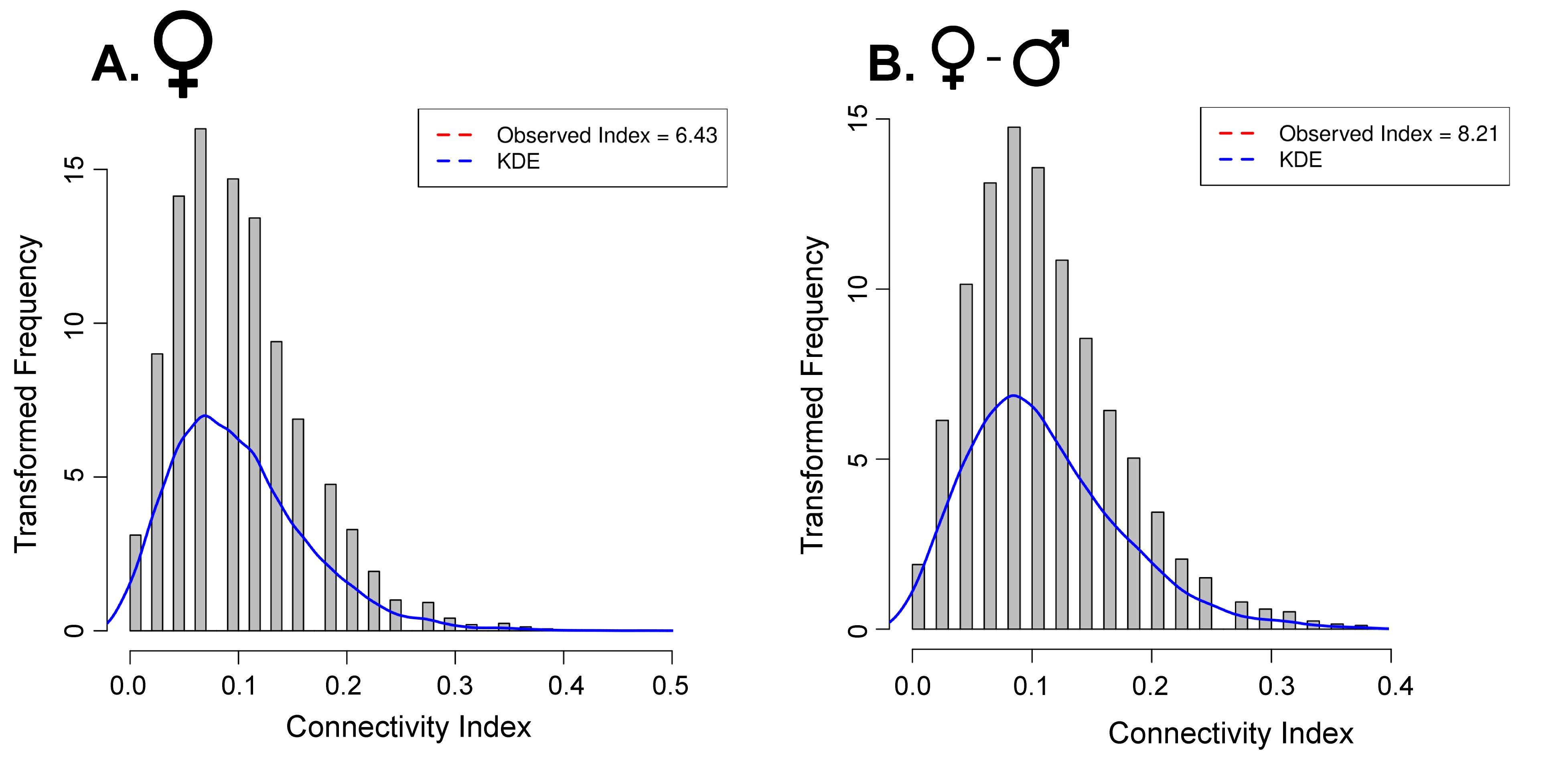

Supplement: Supplementary file 4 — Supplementary Material 4 [file 40246_2024_689_MOESM4_ESM.tif]

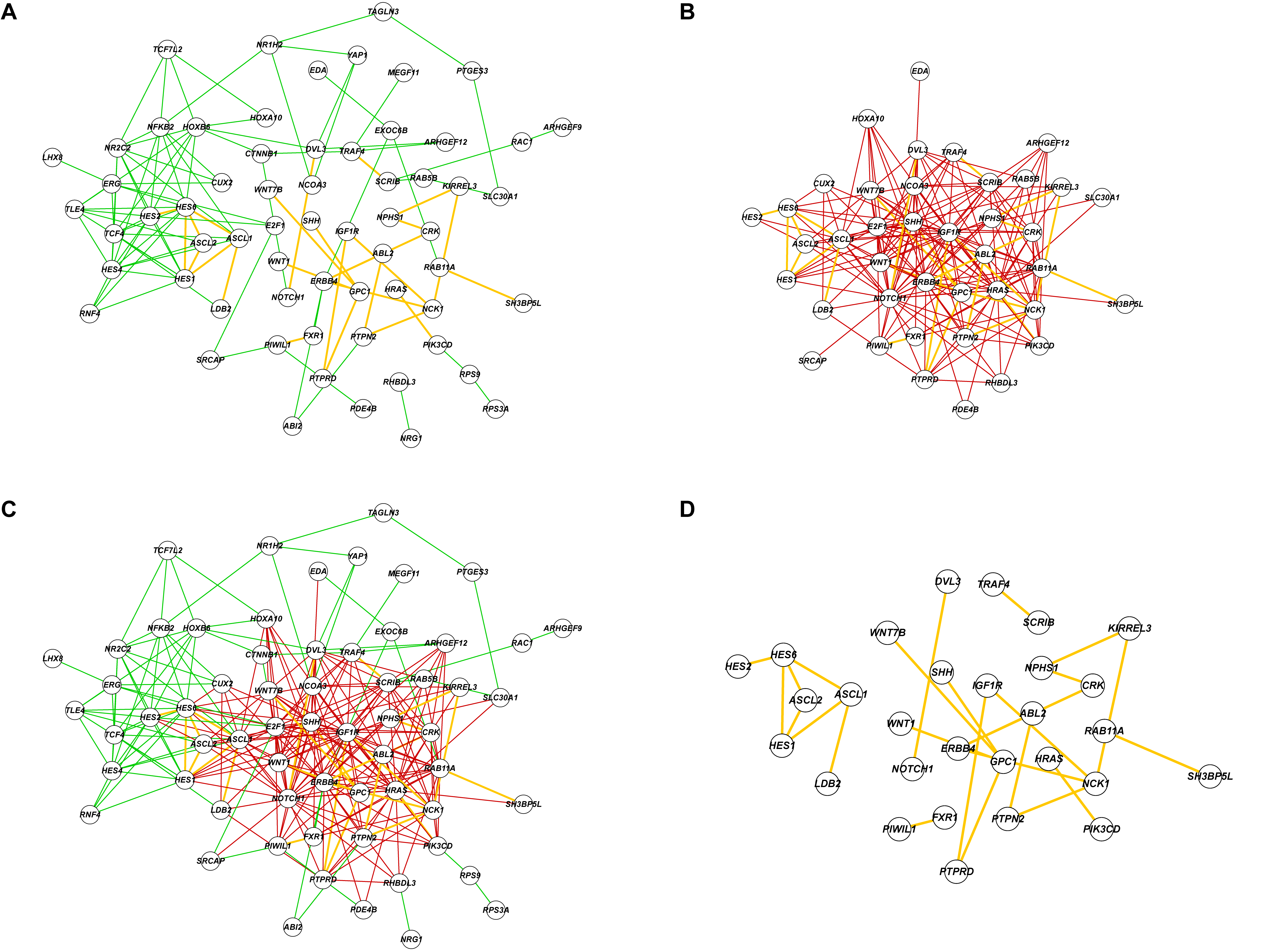

Supplement: Supplementary file 5 — Supplementary Material 5 [file 40246_2024_689_MOESM5_ESM.tif]

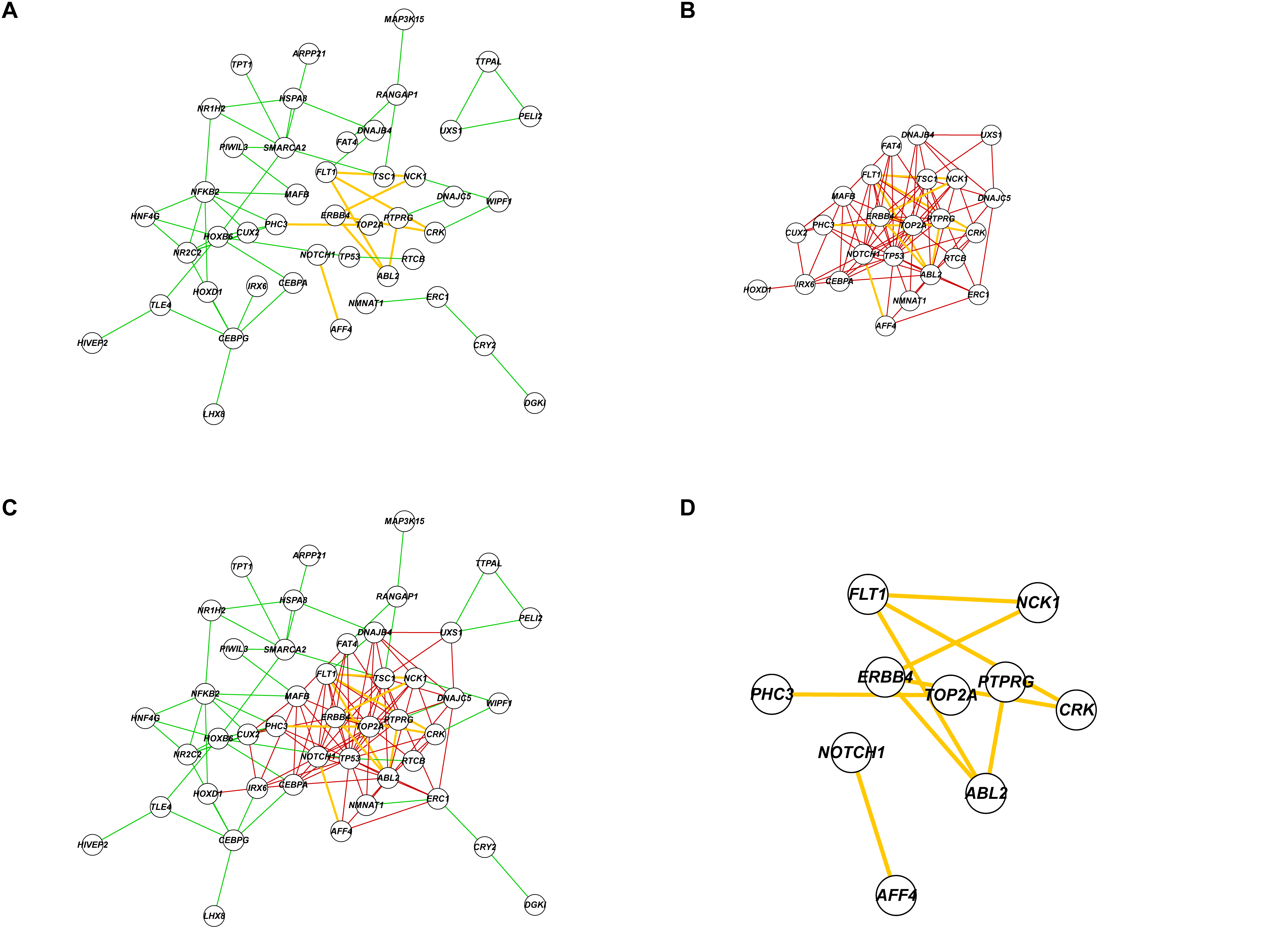

Supplement: Supplementary file 6 — Supplementary Material 6 [file 40246_2024_689_MOESM6_ESM.tif]
